# Dataset of feed bunk score images of cattle feedlot

**DOI:** 10.1016/j.dib.2023.108996

**Published:** 2023-02-21

**Authors:** Brenda Marques de Paula, Gabriel Rezende da Silva, Sabrina Evelin Ferreira, Brian Luís Coimbra Maia, Mathews Edwirds Gomes Almeida, Valdo Martins Soares Júnior, Luiz Maurílio da Silva Maciel, Amália Saturnino Chaves

**Affiliations:** a Programa de Pós-Graduação em Produção Animal, Universidade Federal de Minas Gerais, Montes Claros, MG, Brazil; b Programa de Pós-Graduação em Ciência da Computação, Universidade Federal de Juiz de Fora, Juiz de Fora, MG, Brazil; c Departamento de Medicina Veterinária, Universidade Federal de Juiz de Fora, Juiz de Fora, MG, Brazil; d Departamento de Ciência da Computação, Universidade Federal de Juiz de Fora, Juiz de Fora, MG, Brazil

**Keywords:** Animal nutrition, Precision livestock, Deep learning, Computer vision

## Abstract

Bunk management is an important technique to minimize the variations in consumption in feedlot cattle and can be performed according to the South Dakota State University classification system. The use of information and communication technology (ICT) can help, in an objective way, in the interpretation of these measurements. We created a dataset with the objective to develop an automatic classification method of feed bunk score. In May, September and October on the 2021 and September on the 2022 we captured 1511 images in the morning on the farms, in natural lighting conditions with different angles and backgrounds and at a height of about 1.5 m from the bunk. After acquisition data, each image was classified according to its score classification. Additionally, we resized the images to 500 × 500 pixels, generated annotations files, and organized the dataset in folders*.* The images in this dataset can be used to train and validate a machine learning model to classify feed bunk images. This model can be used to develop an application to support bunk management.

**Specifications Table**

**Table:** 

Subject	Applied Machine Learning, Animal Science, Animal Nutrition, Computer Vision and Pattern Recognition

Specific subject area	Feed Bunk Management, Image Classification
Type of data	Feed bunk score images
How the data were acquired	The data were acquired using a high-resolution rear camera of iPhone 11 (Apple) and a digital camera Cyber-shot DSC—H300 (Sonny). The images were captured on different days, between 6 am and 10 am.
Data format	Raw: .jpg images Annotations: .xml and .csv Distribution: .csv
Description of data collection	The dataset is composed of images of leftover rations on feed bunks in feedlot cattle and represents diet composition according to farms. The main ingredients were silage, citrus pulp and concentrate feed. In this dataset, the feed bunks differ in terms of their material and model. Most feed bunks are made of cement, but some are made of wood with the background covered in rubber or plastic material.
Data source location	The images were collected directly from the feedlot in nine different farms in the state of Minas Gerais, Brazil.
Data accessibility	Repository name: Feed Bunk Score Images - FBSI Data identification number: 10.17632/p9cr67s6jp.1 Direct URL to data: https://data.mendeley.com/datasets/p9cr67s6jp

**Value of the Data**

• This dataset can be useful to develop deep learning algorithms to classify cattle feed images in relation to the amount of leftovers, composition and feed bunk attributes.
• The set of images of leftover feed in bunk can contribute to the development of research on new methods of managing feedlots for confined cattle.
• The methods developed and technological tools obtained through this data can enable farmers and bunk readers to support the management of the animal nutrition and thus optimize the farms’ profits.
• This dataset can be used to explore the potentialities of the applications of computer vision in livestock farming operations.
• This dataset has the potential to develop new tools capable of assisting in the balancing of rations, in the recognition by images of the ingredients used, in the observations of the animals' preference for some food, and reduce costs with cattle feeding.

## 1 Objective

This dataset was created to develop an automatic classification method of feed bunk score. The images could be used to train and validate a machine learning model. This model could be embedded in an application, for mobile devices. In this application the feeder would capture the images using the camera and the application would suggest the adjustments.

## 2 Data Description

The Feed Bunk Score Images - FBSI dataset is a set of images of leftover rations on feed bunks in feedlot cattle. These images are used for reading and classification of the bunk score. This technique is a visual assessment and consists of analyzing the amount of feed left in the bunk over the last 24 h. For each volume of leftovers, specific scores are assigned suggesting adjustments in the amount of feed to be provided during the day, according to the needs of the animals in each herd [1], [2]. In this system, the animals’ residual feed is classified into six bunk scores according to the South Dakota State University (SDSU) score system. For scores 0 and 1/2 it is recommended an increment adjustment, for score 1 a maintenance adjustment, and for scores 2, 3 and 4 a decrement adjustment, according to Table 1 [3]. This visual assessment avoids wasting feed. Since among the operational expenses of the feedlot, feed costs can reach 60% to 80% of the total, avoiding wasting feed is essential for the producers [4].

**Table 1:** Feed bunk scoring SDSU (South Dakota State University).

Score	Features	Adjustment
0	There is no feed in the feed bunk	Increase 1 kg of DM in the diet or 5%
½	Spread food – 5% leftover	Increase 1 kg of DM in the diet or 5%
1	Thin layer (< 5 cm) – 5 to 10% leftover	Keep quantity provided
2	Medium layer (5 to 8 cm) – 25% leftover	Decrease 1 kg of DM in the diet or 5%
3	Thick layer (> 8% cm) – 50% leftover	Decrease 1 kg of DM in the diet or 5% – note other problems
4	Intact food	Decrease 1 kg of DM in the diet or 5% – note other problems

**Fig. 1:**
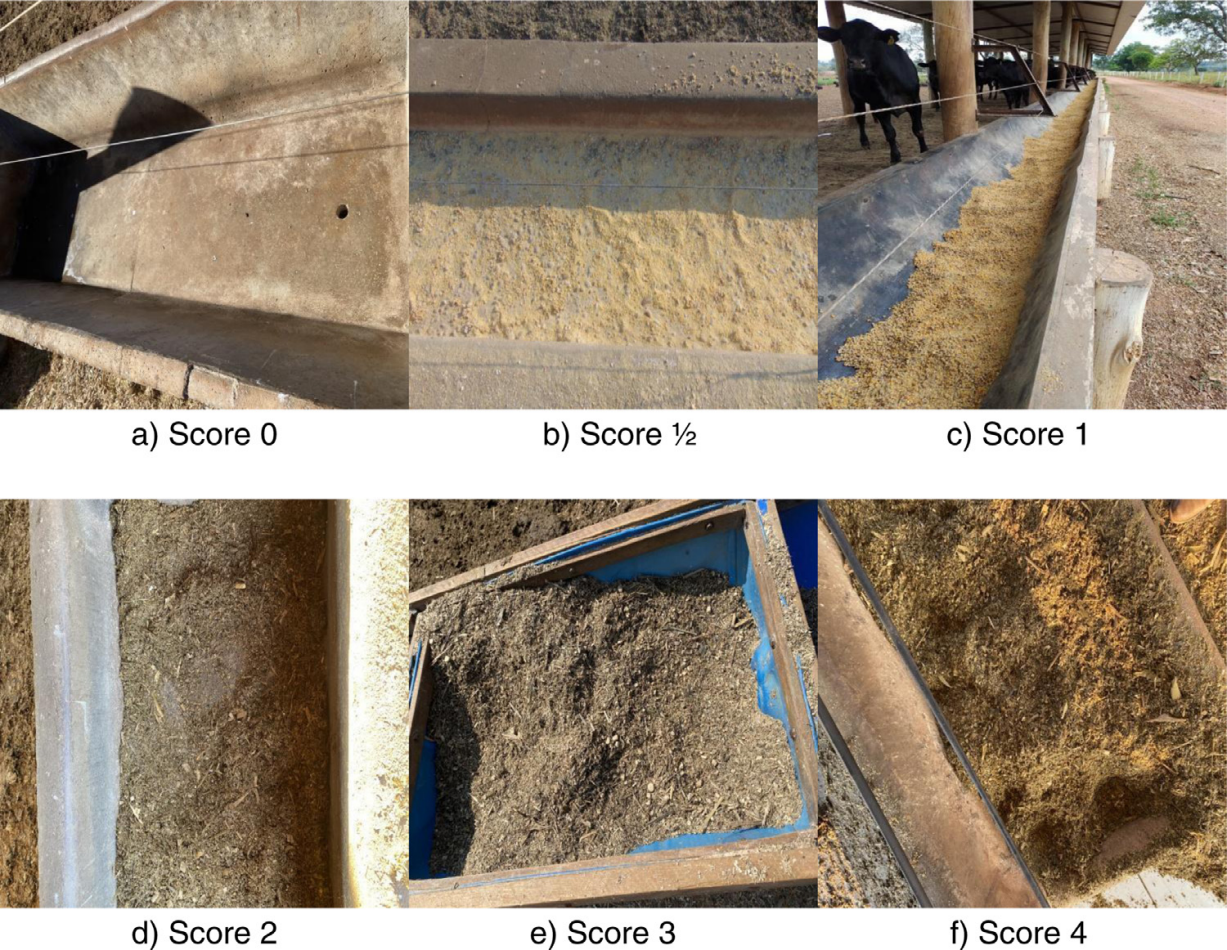
Examples of feed bunks score images.

The dataset is composed of 1511 images of 6 different scores, distributed according to Table 2. These images represent different diet compositions adopted on the farms. Furthermore, in this dataset, the feed bunks differ in terms of their material and model. Most feed bunks are made of cement, but some are made of wood with the background covered in rubber or plastic material. Fig. 1 shows a sample of each score image. Because of the high similarity between classes and high intra-class variability, distinguishing some classes can be a complex task. For these reasons, the images labeling in our dataset was performed by specialists in the animal nutrition field. where:

• Score: represents the score according to Feed Bunk Scoring SDSU system (South Dakota State University).
• Number: represents the image number on the dataset.
• JPG: image format.

**Table 2:** Distribution of the dataset samples, by score.

Score	Amount of samples	Proportion (%)
0	85	5.63
½	266	17.6
1	265	17.54
2	118	7.81
3	270	17.87
4	507	33.55

Each image is uniquely identified by its file name encoded in the format:

We also provide an XML file for each image containing some annotations. Table 3 presents a description and samples of the annotated attributes. For some farms, we could not collect the diet composition data. We indicated this field as “MD” (Missing Data) for these images. Since score 0 represents empty feed bunk, we defined the composition diet field in images of score 0 as “NApp” (Not Applicable). Additionally, we provide a CSV file named “annotations.csv” containing the annotation of all images.

**Table 3:** Annotations in the XML file.

Attribute	Description	Type	Exemple
Name ID	Image identification	Text	score-0_0
Farm	Location where the images were captured	Int	0, 1, 2, etc
Diet composition	Description of the ingredients which the diet is composed	Text	Cornmeal, sorghum, cottonseed, barley
Feed bunk background	Material that composes the feed bunk background	Text	Cement, cement-ceramic, plastic
Format	Geometric form of the feed bunk	Text	H-bunk, J-bunk, feed curb
Adjustment	Suggestion for adjusting the amount of feed supplied to the animals during the day	Text	Increase, maintenance, decrease
Score	Feed bunk scoring according to the leftover feed in the bunk	Real	0, 0.5, 1, 2, 3 and 4

To standardize the comparison between classification methods and mitigate possible biases we suggest a 5–5-fold stratified cross-validation distribution for our dataset. For each distribution and each fold we provide CSV files containing the list of train, test and validation images.

For the purpose of the dataset, we do not provide a bounding box for each image. This is a pre-processing step, which can be performed or not, according to the pipeline designed by everyone who is going to explore the dataset.

## 3 Experimental Design, Materials and Methods

The feed bunk images were acquired using a high-resolution rear camera of iPhone 11 (Apple) and a digital camera Cyber-shot DSC—H300 (Sonny). We also recorded some videos and extracted the frames through Python scripts. To ensure variability, we sampled the videos every 30 frames. The images were collected on different days, between 6 am and 10 am, from eight farms in the state of Minas Gerais, Brazil. We performed the collections in May, September, and October 2021, and September 2022.

The original images had six different spatial resolutions (5152 × 3864, 4608 × 3456, 4032 × 3024, 1280 × 958, 1920 × 1080, and 960 × 1280 pixels) and sRGB color space. We reduced the images to 500 × 500 pixels, which is a suitable resolution for extraction of predictive visual features. Considering the state-of-the-art, several deep convolutional neural networks have input size of 224 × 224 pixels [5], [6], [7]. Another aspect to downscaling image resolutions is the dataset storage scalability, which can become a limitation if dataset updates with addition of new images occur.

To account for variations in environmental conditions, we manually captured the images in natural lighting conditions with different angles and backgrounds and at a height of about 1.5 m from the bunk. In each bunk visited the photos were captured across the entire length. We did not use a static camera that always points to the bunker, but the capture device stood in the hand of the person responsible for the capture, which pointed to the bunker. We collected the images during the daily routine of each farm. Therefore, they are representations of different bunk management and diet composition, and animals of different breeds whose feed consumption is not the same. Thus, this dataset is unbalanced since some scores are more commonly encountered than others. After acquisition data, we classified the images according to feed bunk scoring SDSU [4] and organized them into their respective folders. Table 4 summarizes the steps of data acquisition.

**Table 4:** Data acquisition steps.

No	Steps	Time	Activity
1	Data collecting	May, September and October 2021, and September 2022	The images were captured in the morning, in natural lighting conditions with different angles and backgrounds and at a height of about 1.5 m from the bunk.
2	Images classification	May, September and October, and September 2022	After acquisition data each image was classified according to its score classification.
3	Images processing and dataset organization	October and November 2022	Images were resized to 500 × 500 pixels, XML and CSV files were generated and the dataset organized in folders.

## Ethics Statements

There is no conflict of interest. The data is available in the public domain.

## CRediT authorship contribution statement

**Brenda Marques de Paula:** Conceptualization, Data curation, Writing – original draft, Writing – review & editing. **Gabriel Rezende da Silva:** Data curation, Writing – review & editing, Validation. **Sabrina Evelin Ferreira:** Data curation, Writing – review & editing. **Brian Luís Coimbra Maia:** Data curation, Writing – review & editing, Validation. **Mathews Edwirds Gomes Almeida:** Data curation, Writing – review & editing, Validation. **Valdo Martins Soares Júnior:** Data curation, Writing – review & editing. **Luiz Maurílio da Silva Maciel:** Data curation, Methodology, Supervision, Funding acquisition, Writing – review & editing. **Amália Saturnino Chaves:** Data curation, Methodology, Supervision, Funding acquisition, Writing – review & editing.

## Declaration of Competing Interest

The authors declare that they have no known competing financial interests or personal relationships that could have appeared to influence the work reported in this paper.

## Data Availability

Feed Bunk Score Images - FBSI (Original data) (Mendeley Data).

## References

[1] D.D. Loy, P.J. Gunn, B.E. Doran, R.M. Euken, D.L. Schwab, C.A. Clark, J. Sellers, P.B. Wall, G.R. Dahlke, S. Hoyer, E.L. Lundy, L.L. Schulz, G.A. Dewell, Iowa beef center. Iowa state university animal industry Report, v. 13, 2016. doi:10.31274/ans_air-180814-547.

[2] Luz G.B., de Matos R.F., Cardoso J.B., Brauner C.C. (2019). Exigências nutricionais, cálculos de dieta e mensuração de sobras no manejo nutricional de vacas leiteiras. Pesqui. Agropecu. Gaúch..

[3] D.D. Uyeh, R. Mallipeddi, T. Pamulapati, T. Park, J. Kim, S. Woo, Y. Ha, Interactive livestock feed ration optimization using evolutionary algorithms. Comput. Electron. Agric., v. 155, p. 1–11, 2018. doi:10.1016/j.compag.2018.08.031.

[4] Pritchard R.H., Bruns K.W. (2003). Controlling variation in feed intake through bunk management. J. Anim. Sci..

[5] S. Karen, A. Zisserman, Very deep convolutional networks for large-scale image recognition. arXiv preprint arXiv:1409.1556, 2014

[6] Kaiming H., Zhang X., Ren S., Sun J. (2016). Proceedings of the IEEE Conference on Computer Vision and Pattern Recognition (CVPR).

[7] Huang G., Liu Z., van der Maaten L., Weinberger K.Q. (2017). Proceedings of the IEEE Conference on Computer Vision and Pattern Recognition (CVPR).

